# Prevalence of goiter among children aged 8–10 in Binh Dinh province, Vietnam in 2016–2017

**DOI:** 10.3934/publichealth.2019.2.184

**Published:** 2019-05-23

**Authors:** Truong Quang Dat, Le Nguyen Huong Giang, Pham Van Bao, Nguyen Thi Hong Tuyen

**Affiliations:** 1 Binh Dinh Medical College, Vietnam; 2 Binh Dinh Province's Center for Disease Control and Prevention, Vietnam; 3 Tra Vinh University, Vietnam

**Keywords:** iodine deficiency, goiter, schoolchildren, Binh Dinh, Vietnam

## Abstract

**Objective:**

The study was conducted to estimate the goiter prevalence, and the median urine iodine concentrations among schoolchildren aged 8–10 in Binh Dinh province, Vietnam.

**Methods:**

A school-based cross-sectional survey was carried out from May 2016 to May 2017. A multistage, proportional-to-population-size sampling method with 30 clusters was used. The children were examined by palpation for the presence or absence of goiter based on the criteria of the World Health Organization (WHO), the United Nations Children's Fund (UNICEF), International Council for the Control of Iodine Deficiency (ICCIDD); urinary iodine was determined in microplates by a modification of the Sandell-Kolthoff reaction. The Chi-square test was used to compare prevalences, and the Chi-square test for trend was employed to assess the trend of goiter prevalence and urine iodine levels by age and economic-social areas.

**Results:**

1800 pupils from 8 to 10 years old including 900 males and 900 females were examined and 300 among them were tested for the urinary iodine concentration (UIC). The prevalence of goiter among schoolchildren was 6.6%. The prevalence of goiter tended to increase in areas with disadvantaged conditions, among which the urban areas occupied the lowest prevalence (5%) while the mountainous areas and Midland took the highest (8.8%) (the *p*-value of 0.0193). The median UIC of the study group was 159.9 µg/L; the 25^th^ and 75^th^ percentile value was 103 µg/L and 230.2 µg/L, respectively.

**Conclusion:**

According to the WHO/UNICEF/ICCIDD classification, the goiter prevalence indicated that some regions of Binh Dinh province appeared to be slightly affected by iodine deficiency. These have characterized an important public health challenge, highlighting the need to eliminate iodine deficiency disorders in these areas.

## 1. Introduction

Iodine is one of the vital micronutrient components of the thyroid hormones (thyroxin and triiodothyronine), necessary for normal growth, development, and metabolism during the fetus period, infancy and throughout life [1]. When iodine requirements are not met, thyroid hormone synthesis is impaired, resulting in hypothyroidism and a series of functional and developmental abnormalities grouped under the heading of “Iodine Deficiency Disorders (IDDs)”. Depending on stages of life, the deficiency of iodine has different effects. Iodine deficiency in the fetal stage often causes brain damage such as cretinism, edema and many other harms affecting intellectual, physical, and psychological development. In children and adolescents, the most common manifestation is goiter [2].

Iodine deficiency is a major public health problem for populations throughout the world, particularly for pregnant women and infant children [3]. Globally from 1993–2003, the total goiter prevalence in the general population is estimated to be 15.8%, varying from 4.7% in America to 15.4% in South-East-Asia and 37.3% in Eastern Mediterranean [4].

In 1993, WHO and UNICEF recommended universal salt iodization as the main strategy to achieve elimination of IDDs [5]. This strategy has been implementing in most countries where iodine deficiency is a public health problem. Globally, it is estimated that 75% of households in the world consumed adequately iodized salt [6]. An assessment of global data in 2012 concluded that the number of iodine-deficient countries decreased from 54 to 32 and the number of countries with adequate iodine intake increased from 67 to 105 between 2003 and 2011 as a result of salt iodization [7].

In 1994, the National Iodine Deficiency Disorders Control program was designated as a national priority program in Vietnam, with special funds and oversight [8]. The 2005/2006 national survey indicated that the program enabled Vietnam to achieve the goal of universal salt iodization and eliminate IDDs within a decade in Vietnam. The household coverage of adequately iodized salt increased from 24.9% in 1994 to 93% in 2005/2006, reaching the international goal of over 90%. As a result, the median UIC in children of this age group increased from 14 µg/L in 1998 to 130 µg/L in 2005/2006. The prevalence of goiter among school-age children fell from 12.9% in 1998 to 3.5% in 2005/2006; the global and national targets were to achieve a total goiter prevalence of less than 5% [9]. As the targets had been met, the Government of Vietnam downgraded the National Iodine Deficiency Disorders Control program to be a routine one. As a result of these programmatic changes, there was a dramatic decline in iodized salt coverage and urinary iodine levels to the extent that IDDs had reemerged as a public health problem [10].

Located in the central coast of Vietnam, Binh Dinh has the population of 1,510,350 people in which there are about 122,800 primary schoolchildren all over the province. Binh Dinh's economic development is slow, and the province receives annual subsidies from the national budget [11]. Iodine Deficiency Disorders in Binh Dinh were eliminated in 2005, but the maintaining of this achievement is limited and unsustainable. A survey carried out in 2011 showed that Binh Dinh province had achieved two basic results, namely 92% of households were estimated to consume adequately iodized salt and the prevalence of goiter among children was 3.91%, but the median UIC was low at 93.1 µg/L [12]. Since then, there have not appeared any studies in the whole province for IDDs. We believed that the identification of some indicators of IDDs in Binh Dinh is necessary to know whether iodine deficiency is returning as mentioned in some recent reports. Thus, the present study was designed to identify the goiter prevalence and urinary iodine levels among 8–10-year-old schoolchildren in Binh Dinh Province in 2016–2017.

## 2. Materials and methods

### 2.1. Study design

This was a cross-sectional study based on schools. It was conducted in three socio-economic areas of Binh Dinh province including: (1) urban, (2) coastal plain, and (3) mountainous area and Midland in which the last is the most disadvantageous socio-economic region according to the socio-economic classification of the administrative units directly under the province. The last has the highest percentage of poor households and the lowest per capita incomes [11]. The study was carried out on 8-10-year-old children from May 2016 to May 2017. Participants with thyroid cancer and other thyroid diseases such as infectious thyroiditis, lymphadenopathy were excluded from the study.

### 2.2. Sampling

Sample sizes were calculated using the following formula: $n={\text{Z}}_{1-\text{α}/2}^{2}\frac{\text{p}(1-\text{p})}{{\text{d}}^{2}}$; Where: *n* is the smallest sample size to be achieved; p is the expected prevalence; d is the absolute error; $Z_{1-\alpha /2}$ is Z statistic for a confidence level of 95%.

Sample size for investigating urinary iodine levels: p is the expected prevalence of the mean urine iodine level below 100 µg/L (prevalence of children at the mean urine iodine level below 100 µg/L was 75% [12]; d was the absolute error: 5%. The final sample size was 300 after rounding up.

Sample size for investigating the goiter prevalence: p is the expected prevalence: 5% (a goiter prevalence of 5% or more in schoolchildren is an indication of iodine deficiency in a population) [13]; d is the absolute error: 2.5%. The sample size was adjusted to taking account of the stratification into two genders and three age groups. The final sample size was 1800 after rounding up.

In this study, a total of 1800 children (with 900 males and 900 females) ranging from 8 to 10 years old was surveyed to determine the goiter prevalence and 300 students were tested to determine median urinary iodine levels in all socio-economic regions of Binh Dinh province. Sample sizes were met for analysis.

Sampling method: Multi stage sampling was employed. Stage 1: There are 159 commune-level administrative units (communes, wards, townships) under Binh Dinh Povince as clusters in a sample frame [11]. Thirty clusters were selected using the Probability Proportionate to Size method. One primary school among each cluster was chosen at random. Stage 2: Based on the number of pupils aged 8 to 10 in each class, each cluster of 30 boys and 30 girls (20 students per each age group) was chosen to examine goiter, and then 10 students in every 60 students selected were randomly chosen to collect urine samples to test urinary iodine concentration.

### 2.3. Data collection

Two specialist doctors (for the physical examination) and four nurses were recruited to collect the data.

Variable in goiter was classified using the following categories: grade 0, grade 1 and grade 2. A physical examination was performed at school to identify the presence and absence of goiter in accordance with the criteria of the WHO/UNICEF/ICCIDD [13]. Enlargement of the thyroid gland was assessed by clinical examination, and the goiter was graded as follows: Grade 0: Thyroid gland is neither palpable nor visible/no goiter. Grade 1: A mass in the neck that is consistent with an enlarged thyroid that is palpable but not visible when the neck is in normal position. The mass moves upwards with deglutition/goiter palpable but not visible. Grade 2: A swelling in the neck that is visible when the neck is in normal position and is consistent with enlarged thyroid when the neck is palpated/goiter visible and palpable.

Take urine samples (each sample of 10 mL) to quantify the UIC. The total number of samples was 300. After being collected, urine samples are kept in a closed test tube and refrigerated in an insulated foam container. They were then transported to the laboratory of Nutrition Center of Ho Chi Minh City, which is the leading nutrition center authorized by the Ministry of Health of Vietnam to quantify urinary iodine concentrations in the program of iodine deficiency prevention in Vietnam. Urinary iodine concentrations were determined in micro plates by a modification of the Sandell-Kolthoff reaction. The laboratory clearly defined internal quality control procedures in place, and was opened to external audit. The level of the UIC was classified according to the guidelines of the WHO/UNICEF/ICCIDD [13]. The level of the median UIC lower than 20 µg/L, between 20 µg/L and 49 µg/L, between 50 µg/L, and 99 µg/L indicates the status of iodine deficiency corresponding to a severe, moderate and mild level. The level of the median UIC between 100 µg/L and 199 µg/L shows adequate iodine nutrition; between 200 µg/L and 299 µg/L means the iodine intake is more than necessary, and more than 300 µg/L means excess iodine intake.

### 2.4. Data processing

After cleaning, data were entered into Epi Data 3.1 software and transferred to Stata 10.0 software for data analysis. Absolute values and percentages were used to describe categorical variables. The median, the 25^th^ and 75^th^ percentile values were employed to describe the UIC. The Chi-square test for trend [14] was used to assess the trend of the prevalence of goiter by age and socio-economic areas. Then the Chi-square test was utilized to compare the prevalence in groups. The nonparametric tests (Kruskal-Wallis and Mann-Whitney) to compare the median urine iodine distribution were employed. A *p*-value < 0.05 was considered significant.

### 2.5. Ethical considerations

The study was approved by Binh Dinh Department of Science and Technology. The research was allowed by Education and Training Committee Division in the selected districts and city, and the primary schools at the research sites. All schoolchildren voluntarily participated. The data collected were kept confidential. Students with goiter were advised on treatment. The study results were to be disseminated to relevant stakeholders in order to inform policies and interventions to improve the iodine deficiency status of schoolchildren and paved the way for future studies.

## 3. Results

The data set included the records of 1,800 children (with 900 males and 900 females) aged 8 to 10, among which the Kinh was 93.2% and other ethnic minority groups were 6.8%. Gender distribution for each age group was similar. The size of the samples varied from 840 for the coastal plain area, 540 for the urban area to 420 for the mountainous area and Midland. Similarly, sample sizes by age group from 8 to 10 were 600 for each age group.

As shown in Table 1, children with no goiter, grade 1 and 2 of goiter were 93.4%, 6.3% (95% CI: 5.5–7.8%) and 0.3%, respectively. There was a significant difference between the prevalence of goiter 1 (95% CI: 5.2–7.4%) and goiter 2 (95% CI: 0.1–0.6%).

**Table 1.** The total prevalence of goiter among 8–10-year-old children in Binh Dinh, Vietnam.

Goiter forms	n = 1800	%	95% CI
No goiter	1681	93.4	92.2–94.5
Goiter	119	6.6	5.5–7.8
*Grade 1 of goiter*	*113*	*6.3*	*5.2*–*7.4*
*Grade 2 of goiter*	*6*	*0.3*	*0.1*–*0.6*

Table 2 showed the goiter prevalence according to gender, age groups, ethnic, socio-economic areas. The goiter prevalence among female students was 8.7%, which roughly doubled—the prevalence among male students (*p = 0.0004*). The goiter prevalence varied across ages, in which the prevalence among 9-year-old pupils was 9.2%, which was two times higher than that of 8-year-old students (*p < 0.05*), however, there was no evidence of a trend in the prevalence across age groups (*p = 0.0633*). The goiter prevalence had a tendency to increase; the lowest was in the urban areas (5%), and the highest was in the mountainous areas and Midland (8.8%) with a *p*-value of 0.0193. Nevertheless, the distribution of goiter through socio-economic regions of Binh Dinh province was not different *(p > 0.05)*. The prevalence of goiter among the Kinh (6.3%) was lower than that among other ethnic groups (11.5%) with a *p*-value of 0.025.

Urinary iodine concentration among schoolchildren was presented in Table 3. There were 300 urine samples of students tested, and the median UIC was 159.9 µg/L; the 25^th^ percentile value was 103 µg/L and the 75^th^ percentile value was 230.2 µg/L.

The percentage of urine iodine levels among schoolchildren by socio-economic areas was shown in Table 4. The percentage of the median UIC at the recommended level by the WHO/UNICEF/ICCIDD (100–199 µg/L) in the mountainous area and Midland, the coastal plain area and the urban was 41.1%, 38.6% and 47.1%, respectively but the levels were not associated with socio-economic areas (*p = 0.4916*).

**Table 2.** The goiter prevalence among 8 to 10-year-old children in Binh Dinh, Vietnam according to sex, age, socio-economic areas and ethnic groups.

Variables	Categories	Total	Goiter			*p*
			n	%	95% CI	
Genders	Males	900	41	4.6	3.2–5.9	0.0004
	Females	900	78	8.7	6.8–10.5	
Age	8 years old	600	24	4.0	2.4–5.6	0.0633
	9 years old	600	55	9.2	6.9–11.5	
	10 years old	600	40	6.7	4.8–8.7	
Socio-economic areas	The mountainous area and Midland	420	37	8.8	6.1–11.5	0.0193
	The coastal plain area	840	55	6.5	4.9–8.2	
	The urban area	540	27	5.0	3.2–6.8	
Ethnic groups	The Kinh	1,678	105	6.3	5.1–7.4	0.0250
	Others	122	14	11.5	5.8–17.1	

Note: For gender and ethnic, the *p* is the probability value obtained in the Chi-square test for comparisons of the goiter prevalence between two genders, and between the Kinh and ethnic groups. For ages, socio-economic areas, the *p* is the probability value obtained in the nonparametric test for trends of the goiter prevalence across schoolchildren ages, and three areas.

**Table 3.** The urine iodine concentration among 8–10-year-old children in Binh Dinh, Vietnam according to sex, age, socio-economic areas and ethnic.

Variables	Categories	n	Urine iodine concentration (µg/L)			*p*
			25^th^ percentile	75^th^ percentile	Median	
Genders	Males	148	106.2	244.9	168.5	0.260
	Females	152	99.2	227.3	153.5	
Age	8 years old	97	113.1	232.9	156.5	0.788
	9 years old	102	101.2	224.6	159.4	
	10 years old	101	98.6	246.4	164.9	
Socio-economic areas	The mountainous area and Midland	70	113.1	221.8	159.5	0.338
	The coastal plains area	140	95.8	222.4	157.7	
	The urban area	90	110.9	251.6	165.2	
Ethnic groups	The Kinh	277	107.1	239.6	163.7	0.008
	Others	23	93.1	203.3	107.0	
Total		300	103.0	230.2	159.9	

Note: The *p* is the probability value obtained in the nonparametric test for differences of the median UIC between genders, among age, between ethnic groups and among socio-economic areas.

Table 5 showed the goiter prevalence by the median UIC. The goiter prevalence corresponding to the median urinary iodine level under 100 µg/L (iodine deficiency), from 100 to 199 µg/L, and more was 11.3%, 9.7% and 6.7%, respectively but the *p*-value > 0.05. The data set also showed that there was no evidence of a tendency for the goiter prevalence to go up when urinary iodine levels went down (*p = 0.2796*).

**Table 4.** The percentage of urinary iodine levels by socio-economic areas among 8–10-year-old children in Binh Dinh, Vietnam.

Urinary iodine levels	Median UIC	Total	The urban area (n_u_ = 90)	The coastal plains area (n_c_ = 140)	The mountainous area and midland (n_m_ = 70)	*p*

		No (%)	No (%)	No (%)	No (%)	
< 100 µg/L	75.7 µg/L	71(23.7)	18(20.0)	39(27.9)	14(20.0)	0.8912
100–199 µg/L	150.9 µg/L	124(41.3)	37(41.1)	54(38.6)	33(47.1)	0.4916
≥ 200 µg/L	279.2 µg/L	105(35.0)	35(38.9)	47(33.6)	23(32.9)	0.4053

Note: The *p* is the probability value obtained in the nonparametric test for trends of urine iodine levels across the socio-economic areas.

**Table 5.** The percentage of goiter by urinary iodine levels among 8–10-year-old children in Binh Dinh, Vietnam.

Urinary iodine levels	Total	Median UIC	Goiter			*p*
			n	%	95% CI	
< 100 µg/L	71	75.7 µg/L	8	11.3	3.9–18.6	0.2796
100–199 µg/L	124	150.9 µg/L	12	9.7	4.5–14.9	
≥ 200 µg/L	115	279.2 µg/L	7	6.7	1.7–10.6	

Note: The *p* is the probability value obtained in the nonparametric test for trends of goiter prevalence across of urine iodine levels.

## 4. Discussion

The three most important indicators of impact used in IDDs surveillance are the goiter prevalence, the median UIC, and thyroid function tests [13]. Urinary iodine is the most useful indicator because it is reflective of the current intake of iodine in the diet [15]. To evaluate the severity of IDDs in a region, the most widely accepted marker is the goiter prevalence in schoolchildren. They are the preferred group as it is usually easily accessible. On the basis of the goiter prevalence, the WHO/UNICEF/ICCIDD recommended the criteria for understanding the severity of IDDs as a public health problem in a region. In the study, we observed only the goiter prevalence and the UIC among children aged 8–10.

The present study showed that the overall prevalence of goiter among children from 8 to 10 years old in the study area was 6.6% (95% CI: 5.5–7.8%) in which grade 1 was 6.3% and grade 2 was 0.3%, and it was slightly higher than the cut-off point of the WHO/UNICEF/ICCIDD classification (5%) [13]. However, the prevalence of goiter (6.6%) can be seen as a warning sign for the trend of increasing the goiter prevalence in the study population. This prevalence was in agreement with results reported from a study in Binh Duong, Vietnam in which the general prevalence of goiter was 6.8% [16]. The prevalence of goiter in Binh Dinh is lower than that of the whole country as reported by the National Endocrinology Hospital (9.8%) [17] but higher than those in Binh Dinh over the previous years (3.91%) [12], in Nam Giang district, Quang Nam province, Vietnam (3.2%) [18]. This prevalence in the current study is much lower than those in the studies done in some countries. For example, the goiter prevalence was found to be 62.1% in northeast Ethiopia [19], 77% in Khoramabad, Iran [20], while the prevalence in Shendi, northern Sudan and Belgaum city, India was 14.6% [21] and 16.7% [22], respectively. The reason for the above-mentioned difference may be due to the fact that our study was undertaken after the universal salt iodization launched in Binh Dinh, Vietnam in 1993 [12].

The prevalence of goiter among male students (4.6%) was much lower than that among female students (8.7%). This finding is almost consistent with earlier reports [22]–[25]. This may be due to differences in the developmental stages between males and females. That is, females start puberty earlier than males and require more iodine so girls could be more sensitive to iodine deficiency than boys. In contrast, some studies did not document this difference between boys and girls [18],[26],[27].

In this study, the goiter prevalence ranged from 4% among 8-year-old students to 9.2% among 9-year-old ones. The prevalence of goiter is significantly higher among participants aged 9 than participants aged 8 but the data did not show a trend of the goiter prevalence across age groups. Some studies noted that there is no association between age and goiter [16],[18].

This study also revealed that the goiter prevalence in the urban areas was 5%, but the prevalence tended to go up in the mountainous areas and Midland (8.8%). The high prevalence of goiter in the areas reflects the limited intake of iodine in children in this area. There are several reasons for the highest prevalence of goiter in the mountainous areas and Midland. Soil in these two areas having a limited amount of iodine is the first reason [11],[13],[28]. Another one is that the access to iodized salt of the population in these regions may be inadequate because the place is far from the center of the province, so the transportation, the sale network of iodized salt are very difficult [11],[12]. The proportion of the Kinh (6.3%) suffering from goiter is only about half in comparison with that of other ethnic minority groups (11.5%). This difference can be partly explained by the fact that other ethnic minority groups live in remote and mountainous areas as mentioned above. A recent study on nutrition of children from 6 to 10 years old in Binh Dinh province in 2016 also noted that the malnutrition status among children in the mountainous areas and Midland was the highest among the three social-economic regions of the province [29]. It is possible that this condition is accompanied by a lack of micronutrient deficiency, including iodine.

The median UIC is used as a valuable indicator for the assessment of IDDs in a region. In children, the median UIC of between 100 µg/L and 299 µg/L and not more than 20% of samples below 50 µg/L defines a population which has no iodine deficiency [13]. In the current study, the median UIC is 159.9 µg/L and only 6.7% of samples below 50 µg/L. Therefore, the studied region is not biochemically iodine deficient at present. The median UIC is also much higher than 93.1 µg/L which was the result of the 2011 survey in Binh Dinh [12]. Authors of other studies from Vietnam or other countries indicated different median urinary iodine levels, which pointed to either a deficiency or no deficiency for certain populations, in their areas. Studies in India, Nepal, and Cambodia showed that median UIC was 96 µg/L [25], 93.5 µg/L [30], and 63 µg/L [31], respectively, all of which were below the recommended levels [13]. A study in China documented a 173.3 µg/L median urinary iodine level [32] while a study in Saudi Arabia reported 421 µg/L [33]. Some studies also showed adverse effects of excessive iodine intake [13],[34],[35]. This finding highlights the importance of monitoring the iodine concentration in local food salt and other sources, and not only the median UIC and the prevalence of goiter among schoolchildren.

The median UIC was recorded with no difference between males and females, among age groups and socio-economic regions in the study. This result is consistent with the one in a study in Binh Duong province, Vietnam in which there was no difference in the median UIC by to age and gender [16]. However, the median UIC is significantly higher among the Kinh (163.7 µg/L) than that among other ethnic groups (107 µg/L) but the urine samples in the ethnic groups were relatively small. This difference is in-line with the difference mentioned in the goiter prevalence between the Kinh and other ethnic groups.

In our study, the total goiter prevalence was 6.6%; it was slightly higher than the recommended level by the WHO/UNICEF/ICCIDD, but with the data set recorded in the study, we did not have evidence of the relationship between urinary iodine levels and the goiter prevalence (*p* = 0.2796). It is reported that thyroid size may not return to normal for months or years after the correction of iodine deficiency [13]. Some studies in different countries also did not find the association between urinary iodine levels and the goiter prevalence [25],[36].

Study limitations: The study has some limitations. Firstly, the determination of goiter based only on palpation (without using ultrasonography) may not be objective and depends on the skill of the physicians. Secondly, the proportion of households with iodized salt was not provided in this study. Thirdly, there are certainly some other factors related to the goiter prevalence and urinary iodine levels in Binh Dinh but not yet clarified in this study.

## 5. Conclusion

It is quite clear that the population is not biochemically iodine deficient at present, and the overall prevalence of goiter is found to be around the recommended level by the WHO/UNICEF/ICCIDD. However, the goiter prevalence in mountainous areas and Midland was a warning sign of the public health problem. These have characterized an important public health challenge, highlighting the need to eliminate IDDs in these areas.
